# Bimanual motor impairments in older adults: an updated systematic review and meta-analysis

**DOI:** 10.17179/excli2022-5236

**Published:** 2022-08-16

**Authors:** Nyeonju Kang, Do Kyung Ko, James H. Cauraugh

**Affiliations:** 1 Division of Sport Science, Health Promotion Center, & Sport Science Institute, Incheon National University, Incheon, South Korea; 2 Neuromechanical Rehabilitation Research Laboratory, Incheon National University, Incheon, South Korea; 3 Motor Behavior Laboratory, Department of Applied Physiology and Kinesiology, University of Florida, Gainesville, Florida, USA

**Keywords:** aging, bimanual movement, motor impairment, meta-analysis

## Abstract

This updated systematic review and meta-analysis further examined potential effects of aging on bimanual movements. Forty-seven qualified studies that compared bimanual motor performances between elderly and younger adults were included in this meta-analysis. Moderator variable analyses additionally determined whether altered bimanual motor performances in older adults were different based on the task types (i.e., symmetry vs. asymmetry vs. complex) or outcome measures (i.e., accuracy vs. variability vs. movement time). The random effects model meta-analysis on 80 comparisons from 47 included studies revealed significant negative overall effects indicating more bimanual movement impairments in the elderly adults than younger adults. Moderator variable analyses found that older adults showed more deficits in asymmetrical bimanual movement tasks than symmetrical and complex tasks, and the bimanual movement impairments in the elderly adults included less accurate, more variable, and greater movement execution time than younger adults. These findings suggest that rehabilitation programs for improving motor actions in older adults are necessary to focus on functional recovery of interlimb motor control including advanced motor performances as well coordination.

## Introduction

Voluntary motor actions are essential to our existence. People readily execute motor actions on demand while performing movements required in activities of daily living. Beyond moving our dominant or nondominant arm separately, motor actions often involve both arms moving simultaneously or in synchrony. Coordinating the motor actions of both arms activates movement synergies to meet task demands. Indeed, bimanual movement coordination ranges from rigid and clumsy movements of a novice pianist to elegant and graceful movements of a concert pianist. Moreover, moving both arms in synchrony captures many of the motor actions seen in daily living. For instance, activating simultaneous movements in our left and right arms/hands includes pouring a glass of milk while holding a gallon container in both hands as well as driving a car in a straight line (in the proper lane) while gripping the steering wheel with both hands.

A leading question about motor synergies (i.e., group of muscles that function together as one unit) concerns the effect of aging (Bernshteĭn, 1967[4]). As people reach an elderly age (approximately 65 years old), what happens to their synergies or bimanual movement capabilities? Are they able to execute movements with their left and right arms simultaneously? What about initiating motor actions on stimulus presentation? Once people begin executing bimanual movements are they able to successfully perform correct and consistent motor actions? Are symmetrical movements more resistant to dysfunctional actions than asymmetrical movements? Answers to these questions are important for making informed decisions about an aging population and potential bimanual movement training protocols.

Investigating motor synergies in the elderly deserves full examination given the distinct number of age-related bimanual movement coordination studies recently published (N = 33). Specifically, current research on the elderly extends beyond movements performed by one arm/hand, cognition, posture, and gait. Importantly, planning and executing voluntary movements in both arms simultaneously is consistent with the postulate that activity-based experiences cause new neural connections (Carson, 2005[13]; Cauraugh and Summers, 2005[15]; Cauraugh and Kang, 2021[14]; Rosjat et al., 2018[58]). Intentionally planning and executing coordinated movements with both arms may help the elderly maintain neural plasticity (Cauraugh and Kang, 2021[14]).

Multiple studies reported motor impairment findings in older adults when performing bimanual movements: (a) slower reaction and movement times, (b) decreased movement accuracy, (c) increased variability, and (d) less force production (Jin et al., 2019[40]; Lee et al., 2002[49]; Maes et al., 2021[53]; Wishart et al., 2002[76]). However, other studies did not find bimanual movement differences when comparing older and younger adults (Gorniak and Alberts, 2013[32]; Gulde et al., 2019[34]; Hesse et al., 2020[35]; Kim et al., 2017[41]). These conflicting results lead us to a meta-analysis on aging and bimanual movements (Krehbiel et al., 2017[45]). Three primary findings showed that elderly participants performed bimanual movements with less accuracy, more variability, and slower execution times than younger participants. 

Given the increased research interest in aging and bimanual movements, we wanted to update our earlier findings. Moreover, an additional compelling reason for this systematic review and meta-analysis is to further advance our understanding of motor synergies and bimanual movements in the elderly.

## Material and Methods

### Study identification procedures

According to the PICO suggestion (Cumpston et al., 2022[21]), we set following inclusion criteria: (a) Population: healthy older adults, (b) Intervention: age ≥ 60 years old, (c) Comparator: healthy young adults, and (d) Outcome: quantitative variables indicating bimanual motor performances. Based on these criteria, our literature search focused on potential different bimanual motor performances between healthy older and younger adults. The systematic review and meta-analysis procedures were consistent with the guidelines of the Preferred Reporting Items for Systematic Review and Meta-analysis (PRISMA) (Page et al., 2021[55]). We performed the literature search from May 1, 2022 to June 1, 2022 using two search engines: (1) PubMed and (2) Web of Science. The search keywords based on Boolean logic included: (old OR older OR elderly) AND (bimanual OR bilateral OR interlimb) AND (motor OR movement OR motor control OR force control OR coordination). Four exclusion criteria were observed: (1) studies that reported no relevant quantitative data on bimanual motor performances, (2) animal studies, (3) case studies, and (4) review studies.

### Outcome measures

Given that the current study examined potential altered bimanual motor functions in older adults, we focused on all behavioral data during bimanual motor performance tasks. We categorized bimanual motor performance tasks with four different movement types: (a) symmetrical bimanual movements, (b) asymmetrical bimanual movements, and (c) complex (symmetrical + asymmetrical) bimanual movements. Moreover, we specified bimanual motor functions based on three different perspectives: (a) movement accuracy, (b) movement variability, and (c) movement time. 

### Meta-analytic approaches

All meta-analysis procedures were conducted using the Comprehensive Meta-Analysis software (ver. 3.3, Englewood, NJ, USA). Consistent with conventional methodology (Borenstein et al., 2009[10]), we calculated individual and overall effect sizes using the standardized mean difference (*SMD*) with 95 % confidence intervals. More negative values of effect sizes indicated that older adults revealed greater impairments in bimanual motor performances (e.g., more erroneous and variable movement and greater movement time) than those in younger adults. Further, we used random effects meta-analysis models for synthesizing individual effect sizes because the included studies used different characteristics of participants, outcome measures, or bimanual motor tasks (Borenstein et al., 2010[10]). Moreover, we conducted moderator variable analyses for examining two specific sub-questions: (a) Do bimanual motor performances in older adults differ among characteristics of task (i.e., symmetry vs. asymmetry vs. complex)? and (b) Do bimanual motor performances in older adults differ among characteristics of outcome measures (i.e., accuracy vs. variability vs. movement execution time)?

To quantify variability of effect sizes across the included studies, we performed two heterogeneity tests: (a) Cochrane's *Q* and *P*-value and (b) the Higgins and Green *I*^2^. Cochrane's *Q* is based on the chi square distribution so that *Q*-statistics with *P*-value greater than 0.05 indicates significant heterogeneity between individual effect sizes (Borenstein et al., 2009[10]). The values of *I*^2^ that exceed 75 % denote high level of heterogeneity (Higgins et al., 2003[36]). Regarding the publication bias assessment that shows asymmetry of individual effect sizes, the Egger regression test was used. This approach provides intercept (β_0_) and *P*-value so that greater *P*-value than 0.05 indicates significant publication bias (Egger et al., 1997[24]). 

## Results

### Qualified studies for the meta-analysis

**Figure 1:**
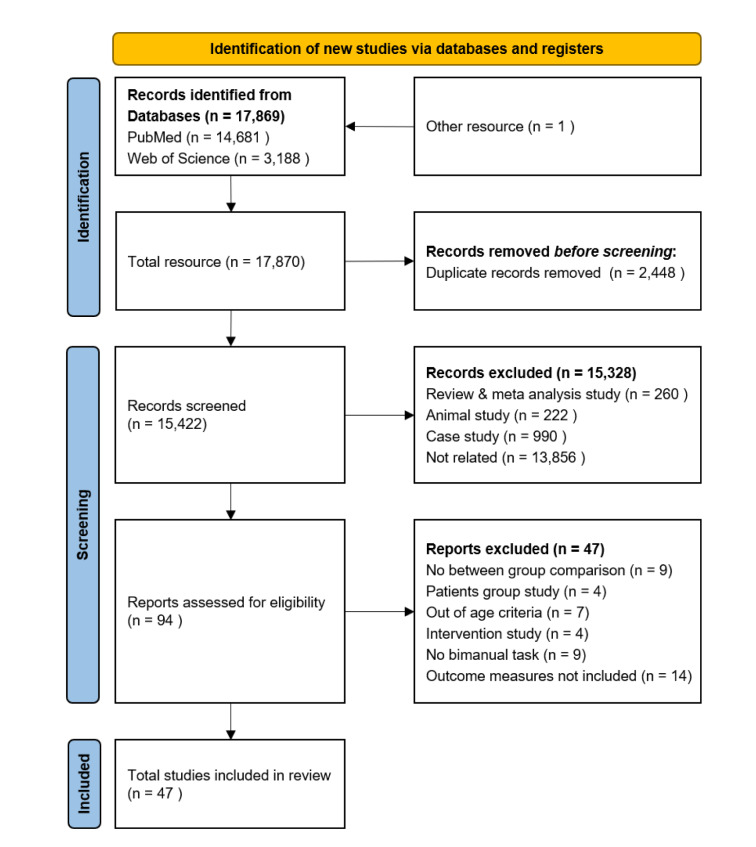
PRISMA flow chart describing study identification procedures

Our literature search identified 17,870 potential articles from two search engines. After removing 2,448 duplicated articles, the title and abstract for 15,422 studies were firstly screened. We excluded 15,328 articles because of 260 review articles, 222 animal studies, 990 case studies, and 13,856 studies that focused on a different research topic. The remaining 94 articles were fully reviewed. Further, we decided to exclude 47 articles that did not meet our inclusion criteria. Finally, 47 studies qualified for this meta-analysis (Addamo et al., 2010[1]; Babaeeghazvini et al., 2019[2]; Bangert et al., 2010[3]; Bhakuni and Mutha, 2015[5]; Blais et al., 2014[6]; Boisgontier et al., 2014[8]; Boisgontier and Swinnen, 2015[7]; Britten et al., 2017[11]; Coats and Wann, 2012[16]; Coffman et al., 2021[17]; Coxon et al., 2010[20]; Dickins et al., 2017[23]; Fling and Seidler, 2012[25]; Fling et al., 2011[26]; Fujiyama et al., 2016[28]; Goble et al., 2010[30]; Gorniak and Alberts, 2013[32]; Gulde and Hermsdörfer, 2017[33]; Gulde et al., 2019[34]; Hesse et al., 2020[35]; Hu and Newell, 2011[37]; Jin et al., 2019[40]; Kim et al., 2017[41]; King et al., 2018[42]; Kiyama et al., 2014[43]; Kornatz et al., 2021[44]; Lee et al., 2002[49]; Loehrer et al., 2016[51]; Maes et al., 2021[53]; Monteiro et al., 2019[54]; Pauwels et al., 2018[56]; Rudisch et al., 2020[59]; Rueda-Delgado et al., 2019[60]; Sallard et al., 2014[62]; Santos Monteiro et al., 2017[63]; Seer et al., 2021[64]; Serbruyns et al., 2015[65]; Serrien et al., 2000[66]; Solesio-Jofre et al., 2018[67]; Stelmach et al., 1988[69]; Summers et al., 2010[70]; Swinnen, 1998[71]; Temprado et al., 2010[74], 2020[73]; Wishart et al., 2000[77], 2002[76]; Zivari Adab et al., 2018[78]). Specific procedures for the study identification are shown in the PRISMA flow chart (Figure 1(Fig. 1)).

### Participant characteristics

A total of 965 older adults (mean age and SD = 69.4±5.4 years, 515 females) and 878 younger adults (mean and SD of age = 23.2±2.9 years, 450 females) participated in the qualified studies. We confirmed that all included studies recruited healthy older and younger adults without any neurological disorder and musculoskeletal impairments in their upper extremities. Table 1(Tab. 1) (References in Table 1: Addamo, 2010[1]; Babaeeghazvini, 2019[2]; Bangert, 2010[3]; Bhakuni, 2015[5]; Blais, 2014[6]; Boisgontier, 2014[8], 2015[7]; Britten, 2017[11]; Coats, 2012[16]; Coffman, 2021[17]; Coxon, 2010[20]; Dickins, 2017[23]; Fling, 2011[26], 2012[25]; Fujiyama, 2016[28]; Goble, 2010[30]; Gorniak, 2013[32]; Gulde, 2017[33], 2019[34]; Hesse, 2020[35]; Hu, 2011[37]; Jin, 2019[40]; Kim, 2017[41]; King, 2018[42]; Kiyama, 2014[43]; Kornatz, 2021[44]; Lee, 2002[49]; Loehrer, 2016[51]; Maes, 2021[53]; Monteiro, 2019[54]; Pauwels, 2018[56]; Rudisch, 2020[59]; Rueda-Delgado, 2019[60]; Sallard, 2014[62]; Santos Monteiro, 2017[63]; Seer, 2021[64]; Serbruyns, 2015[65]; Serrien, 2000[66]; Solesio-Jofre, 2018;[67] Stelmach, 1988[69]; Summers, 2010[70]; Swinnen, 1998[71]; Temprado, 2010[74], 2020[73]; Wishart, 2000[77], 2002[76]; Zivari Adab, 2018[78]) shows specific demographic information.

### Bimanual motor performance variables

The 47 included studies used 48 bimanual motor performance tasks: (a) tracking task: 11 studies, (b) force control task: five studies, (c) tapping task: 10 studies, (d) cyclical movements task: 11 studies, (e) reaching task: four studies, (f) grip or grasp task: two studies, (g) activity of daily living task: two studies, (h) reaction task: two studies, and (i) matching task: one study. In addition, the 48 bimanual motor performance tasks involved 56 detailed comparisons based on task symmetry: (a) 13 symmetrical task comparisons, (b) 16 asymmetrical task comparisons, and (c) three complex (symmetrical + asymmetrical) task comparisons. Unfortunately, 24 task comparisons included combined behavioral data from separate symmetrical and asymmetrical bimanual motor performance tasks. Finally, to compare bimanual motor performances between older and younger adult groups, we found 41 accuracy comparisons from 34 studies that estimated motor accuracy, 21 variability comparisons from 19 studies that assessed motor variability, and 18 movement time comparisons from 15 studies that measured movement time. Specific details on bimanual motor performance tasks used for the included studies are shown in Table 2(Tab. 2) (References in Table 2: Addamo, 2010[1]; Babaeeghazvini, 2019[2]; Bangert, 2010[3]; Bhakuni, 2015[5]; Blais, 2014[6]; Boisgontier, 2014[8], 2015[7]; Britten, 2017[11]; Coats, 2012[16]; Coffman, 2021[17]; Coxon, 2010[20]; Dickins, 2017[23]; Fling, 2011[26], 2012[25]; Fujiyama, 2016[28]; Goble, 2010[30]; Gorniak, 2013[32]; Gulde, 2017[33], 2019[34]; Hesse, 2020[35]; Hu, 2011[37]; Jin, 2019[40]; Kim, 2017[41]; King, 2018[42]; Kiyama, 2014[43]; Kornatz, 2021[44]; Lee, 2002[49]; Loehrer, 2016[51]; Maes, 2021[53]; Monteiro, 2019[54]; Pauwels, 2018[56]; Rudisch, 2020[59]; Rueda-Delgado, 2019[60]; Sallard, 2014[62]; Santos Monteiro, 2017[63]; Seer, 2021[64]; Serbruyns, 2015[65]; Serrien, 2000[66]; Solesio-Jofre, 2018[67]; Stelmach, 1988[69]; Summers, 2010[70]; Swinnen, 1998[71]; Temprado, 2010[74], 2020[73]; Wishart, 2000[77], 2002[76]; Zivari Adab, 2018[78]).

### Meta-analysis findings: overall effects

**Figure 2:**
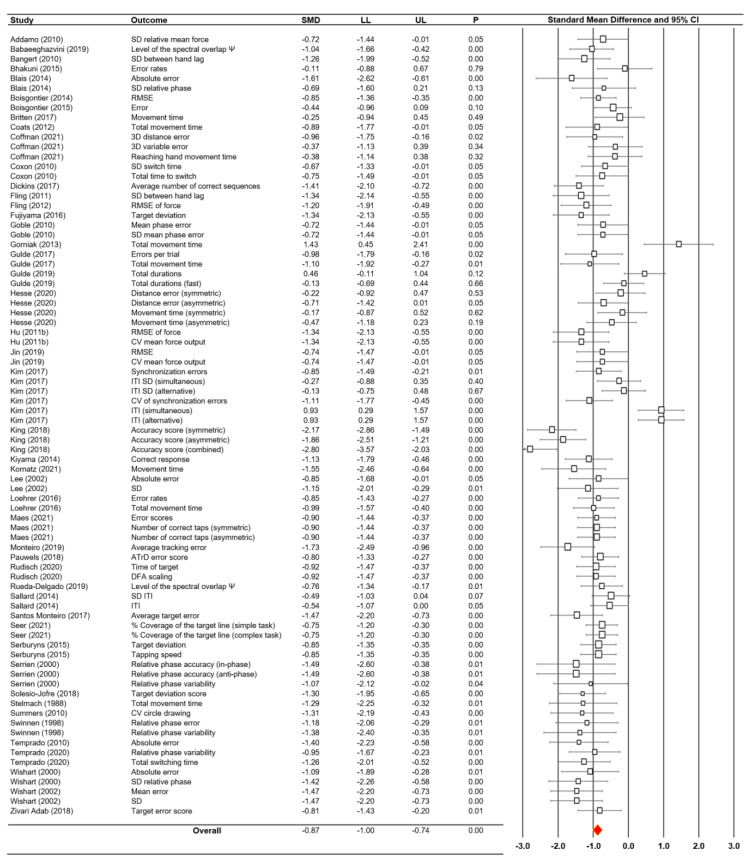
Meta-analysis findings and forest plot for bimanual motor performance in older adults versus younger adults

A random effects model meta-analysis conducted on 80 total comparisons from 47 studies revealed significant overall effects indicating bimanual motor impairments in the older adults when compared to motor actions of younger adults (*SMD* = -0.87; *SE* = 0.07; 95 % CI = -1.00 - -0.74*; Z* = −12.97; *P* < 0.001; Figure 2(Fig. 2); References in Figure 2: Addamo, 2010[1]; Babaeeghazvini, 2019[2]; Bangert, 2010[3]; Bhakuni, 2015[5]; Blais, 2014[6]; Boisgontier, 2014[8], 2015[7]; Britten, 2017[11]; Coats, 2012[16]; Coffman, 2021[17]; Coxon, 2010[20]; Dickins, 2017;[23] Fling, 2011[26], 2012[25]; Fujiyama, 2016[28]; Goble, 2010[30]; Gorniak, 2013[32]; Gulde, 2017[33], 2019[34]; Hesse, 2020[35]; Hu, 2011[37]; Jin, 2019[40]; Kim, 2017[41]; King, 2018[42]; Kiyama, 2014[43]; Kornatz, 2021[44]; Lee, 2002[49]; Loehrer, 2016[51]; Maes, 2021[53]; Monteiro, 2019[54]; Pauwels, 2018[56]; Rudisch, 2020[59]; Rueda-Delgado, 2019[60]; Sallard, 2014[62]; Santos Monteiro, 2017[63]; Seer, 2021[64]; Serbruyns, 2015[65]; Serrien, 2000[66]; Solesio-Jofre, 2018[67]; Stelmach, 1988[69]; Summers, 2010[70]; Swinnen, 1998[71]; Temprado, 2010[74], 2020[73]; Wishart, 2000[77], 2002[76]; Zivari Adab, 2018[78]). These findings indicated a relatively large negative effect (≥ 0.80) (Cohen, 1988[18]; Rosenthal and DiMatteo, 2001[57]). Furthermore, given that six individual effect sizes from four studies (Gorniak and Alberts, 2013[32]; Gulde et al., 2019[34]; Kim et al., 2017[41]; King et al., 2018[42]) exceeded two standard deviations beyond the mean of individual effect sizes, we conducted a sensitivity analysis after removing these six potential outliers. The analysis confirmed that the standardized effect was comparable to the original effect size (*ES* = -0.89; *SE *= 0.05; 95 % CI = -0.98 - -0.81; *Z* = −19.64; *P* < 0.001). These findings indicate that the older adults showed more impairments in bimanual motor performances than the younger adults.

The heterogeneity tests revealed moderate levels of variability across the included studies: (a) Cochrane's *Q* = 230.99, *P* < 0.001 and (b) Higgins and Green's *I*^2^ = 65.80 %. However, an additional heterogeneity test conducted after removing the six potential outliers revealed minimal levels of variability across the included studies: (a) Cochrane's *Q* = 92.30, *P* = 0.06 and (b) Higgins and Green's *I*^2^ = 20.91 %. For estimating potential publication bias, Egger's regression analysis showed a significant intercept (*β*_0_ = −2.14; *P* = 0.022) indicating potential asymmetrical distribution of individual effect sizes. An additional Egger's regression test conducted after removing six potential outliers revealed a significant intercept (*β*_0_ = −2.17; *P* < 0.001).

### Moderator variable analyses: task symmetry vs. asymmetry vs. complex

**Figure 3:**
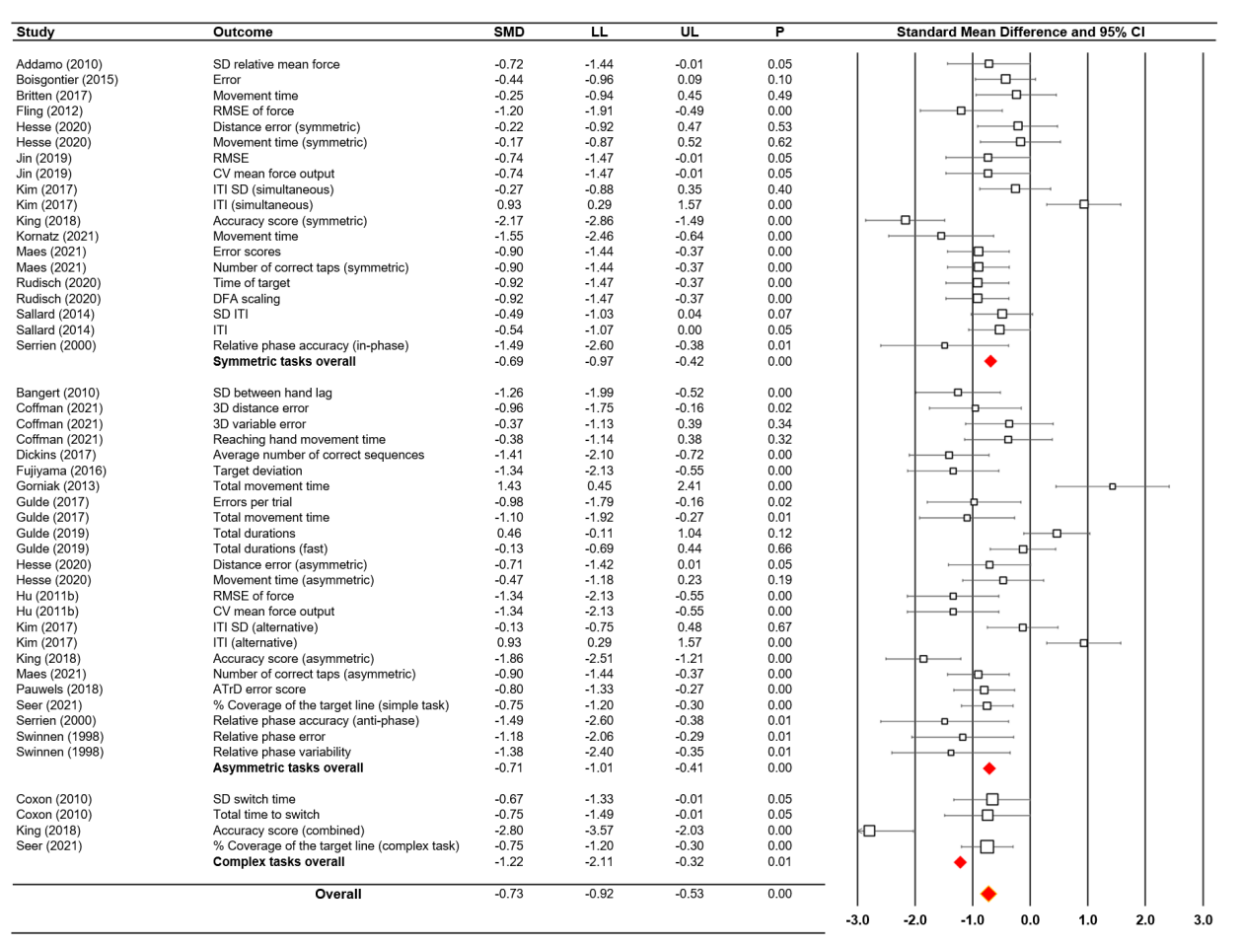
Moderator variable analysis findings on different task types and forest plot for bimanual motor performance in older adults versus younger adults

Moderator variable analyses examined three types of bimanual motor performance tasks to further explore the data: (a) symmetric tasks, (b) asymmetric tasks, and (c) complex tasks. Nineteen symmetric task comparisons from 13 studies reported moderate negative effect results: *SMD* = -0.69; *SE *= 0.14; 95 % CI = -0.97 - -0.42; *Z* = −5.00; *P* < 0.001; Cochrane's *Q* = 61.11 and *P* < 0.001; *I*^2^ = 70.54 % (Figure 3(Fig. 3); References in Figure 3: Addamo, 2010[1]; Bangert, 2010[3]; Boisgontier, 2014[8], 2015[7]; Britten, 2017[11]; Coffman, 2021[17]; Coxon, 2010[20]; Dickins, 2017[23]; Fling, 2012[25]; Fujiyama, 2016[28]; Gorniak, 2013[32]; Gulde, 2017[33], 2019[34]; Hesse, 2020[35]; Hu, 2011[37]; Jin, 2019[40]; Kim, 2017[41]; King, 2018[42]; Kornatz, 2021[44]; Maes, 2021[53]; Pauwels, 2018[56]; Rudisch, 2020[59]; Sallard, 2014[62]; Seer, 2021[64]; Serrien, 2000[66]; Swinnen, 1998[71]). In addition, a sensitivity analysis that excluded two potential outliers (Kim et al., 2017[41]; King et al., 2018[42]) revealed a similar negative effect (*SMD* = -0.69; *SE *= 0.09; 95 % CI = -0.85 - -0.52; *Z* = −8.01; *P* < 0.001; Cochrane's *Q* = 19.02 and *P* = 0.27; *I*^2^ = 15.87 %). 

Concerning the asymmetric task, 24 comparisons from 16 studies indicated a moderate negative effect (*SMD* = -0.71; *SE* = 0.15; 95 % CI = -1.01 - -0.41; *Z *= −4.66; *P* < 0.001; Cochrane's *Q* = 100.19 and *P* < 0.001; *I*^2^ = 77.04 %; Figure 3(Fig. 3)). The sensitivity analysis excluded three potential outliers (Gorniak and Alberts, 2013[32]; Gulde et al., 2019[34]; Kim et al., 2017[41]) and revealed a large and negative effect (*SMD* = -0.92; *SE *= 0.11; 95 % CI = -1.13 - -0.71; *Z* = −8.62;* P* < 0.001; Cochrane's *Q* = 36.44 and *P* = 0.014; *I*^2^ = 45.11 %). 

Analysis on four complex task comparisons from three studies reported a relatively large negative effect (*SMD* = -1.22; *SE* = 0.46; 95 % CI = -2.11 - -0.32; *Z *= −2.66; *P* = 0.008; Cochrane's *Q* = 23.72 and *P* < 0.001; *I*^2 ^= 87.35 %; Figure 3(Fig. 3)). The sensitivity analysis, which excluded one potential outlier (King et al., 2018[42]), revealed a moderate and negative effect (*SMD* = -0.73; *SE *= 0.17; 95 % CI = -1.06 - -0.40; *Z* = −4.32;* P* < 0.001; Cochrane's *Q* = 0.04 and *P* = 0.978; *I*^2^ = 00.00 %). 

Overall, these moderator variable findings indicate that older adults showed lower bimanual motor performances across the symmetric, asymmetric, and complex tasks than the younger adults. Moreover, the impairments increased more in the asymmetric bimanual motor tasks than the symmetric and complex moderator variables.

### Moderator variable analysis: motor accuracy vs. motor variability vs. movement execution time

**Figure 4:**
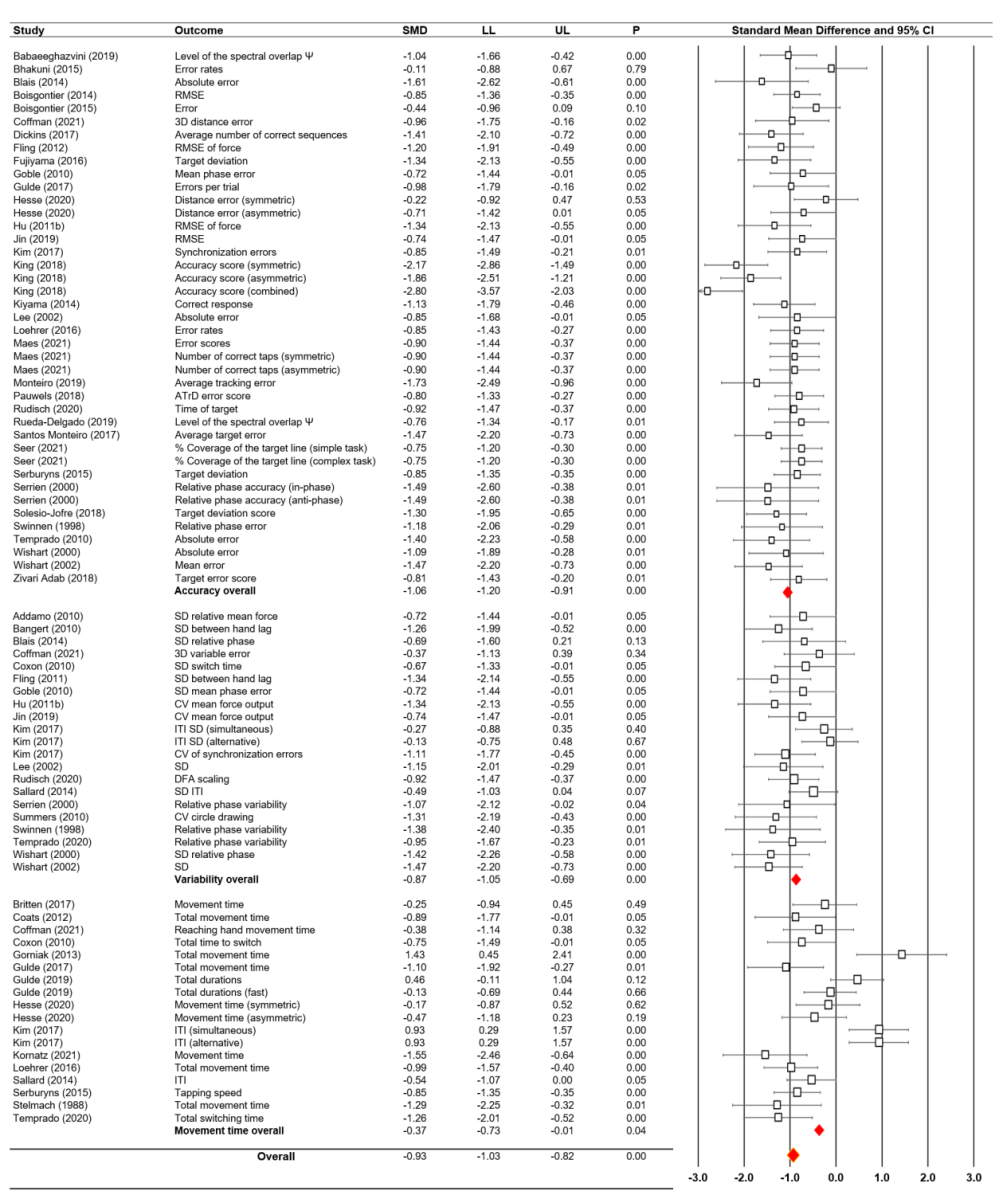
Moderator variable analysis findings on different outcome measures and forest plot for bimanual motor performance in older adults versus younger adults

Our second subgroup meta-analysis examined the potential effects of moderator variables among three types of traditional bimanual motor performance outcome measures: (a) accuracy, (b) variability, and (c) movement execution time. Forty-one accuracy comparisons from 34 studies revealed a large negative effect (*SMD* = -1.06; *SE *= 0.07; 95 % CI = -1.20 - -0.91; *Z* = −14.47; *P* < 0.001; Cochrane's *Q* = 75.59 and *P* = 0.001; *I*^2^ = 47.09 %; Figure 4(Fig. 4); References in Figure 4: Addamo, 2010[1]; Babaeeghazvini, 2019[2]; Bangert, 2010[3]; Bhakuni, 2015[5]; Blais, 2014[6]; Boisgontier, 2014[8], 2015[7]; Britten, 2017[11]; Coats, 2012[16]; Coffman, 2021[17]; Coxon, 2010[20]; Dickins, 2017[23]; Fling, 2011[26], 2012[25]; Fujiyama, 2016[28]; Goble, 2010[30]; Gorniak, 2013[32]; Gulde, 2017[33], 2019[34]; Hesse, 2020[35]; Hu, 2011[37]; Jin, 2019[40]; Kim, 2017[41]; King, 2018[42]; Kiyama, 2014[43]; Kornatz, 2021[44]; Lee, 2002[49]; Loehrer, 2016[51]; Maes, 2021[53]; Monteiro, 2019[54]; Pauwels, 2018[56]; Rudisch, 2020[59]; Rueda-Delgado, 2019[60]; Sallard, 2014[62]; Santos Monteiro, 2017[63]; Seer, 2021[64]; Serbruyns, 2015[65]; Serrien, 2000[66]; Solesio-Jofre, 2018[67]; Stelmach, 1988[69]; Summers, 2010[70]; Swinnen, 1998[71]; Temprado, 2010[74], 2020[73]; Wishart, 2000[77] 2002[76]; Zivari Adab, 2018[78]). In addition, a sensitivity analysis that excluded two potential outliers from one study (King et al., 2018[42]) reported similar negative effect results: *SMD* = -0.96; *SE *= 0.06; 95 % CI = -1.08 - -0.85; *Z* = −17.07; *P* < 0.001; Cochrane's *Q* = 42.58 and *P* = 0.280; *I*^2^ = 10.75 %. 

The analysis on 21 variability comparisons from 19 studies revealed a large negative effect (*SMD* = -0.87; *SE* = 0.09; 95 % CI = -1.05 - -0.69; *Z *= −9.60; *P* < 0.001; Cochrane's *Q* = 24.83 and *P* = 0.208; *I*^2^ = 19.45 %; Figure 4(Fig. 4)). For this variability moderator, the sensitivity analysis was not necessary because there were no outliers. 

Eighteen movement execution time comparisons from 15 studies revealed a relatively small negative effect (*SMD* = -0.37; *SE* = 0.18; 95 % CI = -0.73 - -0.01; *Z *= −2.03; *P* = 0.043; Cochrane's *Q* = 82.07 and *P* < 0.001; *I*^2 ^= 79.29 %; Figure 4(Fig. 4)). The sensitivity analysis, which excluded four potential outliers from three studies (Gorniak and Alberts, 2013[32]; Gulde et al., 2019[34]; Kim et al., 2017[41]), revealed a moderate and negative effect (*SMD* = -0.70; *SE *= 0.11; 95 % CI = -0.92 - -0.48; *Z* = −6.23;* P* < 0.001; Cochrane's *Q* = 18.42 and *P* = 0.142; *I*^2^ = 29.41 %;). 

In summary, these moderator variable findings show that the older adults executed less accurate and more variable bimanual motor performances than the younger participants. Further, the elderly adults required longer movement times to execute the bimanual motor tasks in comparison to the younger adults.

## Discussion

The purpose of this systematic review and meta-analysis was to further investigate the effect of aging on bimanual movements. We found 47 studies that reported young and elderly findings while participants performed bimanual movements. The data from these studies generated 80 comparisons on a total of 1,848 participants (969 elderly) for our random effects model meta-analysis. Our analysis revealed an overall standardized mean difference that showed significantly more bimanual movement impairments in the elderly adults versus younger adults. Further, the older adults revealed more deficits in asymmetrical bimanual movement tasks than symmetrical and complex tasks. Bimanual movement impairments in the elderly adults included less accurate, more variable, and greater movement execution time than younger adults. These robust large negative effects found in the elderly further demonstrates an aging problem involving motor synergies.

Motor synergy differences between old and young people reflect aging changes in the neuromuscular system as well as an apparent tendency in the elderly to execute bimanual motor actions that are safe (Latash, 2008[48]). As people age the neuromuscular system functions less efficiently because of a decrease in the number of cortical neurons as well as fewer alpha-motoneurons that send their axons to muscle fibers (Cordo and Harnad, 1994[19]; Gazzaniga and Mangun, 2014[29]; Latash, 2008[48]; Spirduso et al., 2005[68]). Support for dysfunctional bimanual movements in the elderly is found in our three moderator variable analyses conducted on bimanual movement accuracy, variability, and execution time. Meta-analysis of the 41 accuracy comparisons on the bimanual movements identified a similar effect size as the overall analysis. Elderly adults are less accurate than younger adults when performing bimanual movements. In terms of variability (21 comparisons) and execution time (18 comparisons), elderly people are more variable and take longer to execute bimanual movements than young people. These aging motor synergy impairments highlight some of the bimanual movement challenges reported in other studies. 

Age-related neurodegenerative changes were examined in the motor system using functional MRI (Ward and Frackowiak, 2003[75]). When Ward and Frackowiak found an increase in activity of the caudal dorsal premotor cortex and caudal cingulate sulcus, they interpreted the age-related effect as evidence favoring an adaptable motor system. Some elderly people were able to complete the simple unimanual tasks with increased cortical activation levels. Perhaps the neuroplasticity of the motor system found during unimanual movements could be activated during bimanual movements to minimize age-related impairments. Certainly, bimanual movements activate bilateral interactions in various brain regions: (a) primary motor cortex, (b) supplementary motor area, (c) basal ganglia, and (d) cerebellum (Carson, 2005[13]; Cordo and Harnad, 1994[19]). Further, Swinnen and Wenderoth (2004[72]) postulated that cognitive neuroscience affects bimanual movements. Cognitive input may play a role in the age-related changes in the neuromuscular system while elderly adults perform complex tasks involving bimanual movements (Swinnen and Wenderoth, 2004[72]).

Additional moderator variable analyses on types of bimanual movements required by the tasks produced novel findings. Analysis of 13 studies that tested symmetrical tasks generated 19 comparisons and showed a medium negative effect in the older adults on bimanual symmetrical movements than the younger adults. Further, analysis of the asymmetrical bimanual tasks (16 studies; 24 comparisons) revealed a large negative effect for the elderly versus the young. These findings on the asymmetrical tasks showing more motor action impairments are consistent with the literature (Bangert et al., 2010[3]; Hu and Newell, 2011[38][37]). Indeed, there is a long history of asymmetrical bimanual movements indicating aging motor impairments (i.e., less stable than symmetrical movements) (Bangert et al., 2010[3]; Byblow et al., 1999[12]; Stelmach et al., 1988[69]). The asymmetrical bimanual tasks may require more neural interactions via the corpus callosum between left and right hemispheres to compensate for higher motor variability. Moreover, the integrity of the corpus callosum may be related to impaired bimanual coordination functions (Gooijers and Swinnen, 2014[31]; Hung et al., 2019[39]). Given that structural development and degeneration in the corpus callosum presumably follow an inverted-U-shape trajectory across the lifespan (Danielsen et al., 2020[22]), elderly adults with potential impairments in key brain structures may experience more difficulty with asymmetrical bimanual motor tasks.

In a dynamic systems perspective, Temprado and colleagues reported bimanual coupling iterations across ages (Temprado et al., 2010[74]). Older adults performed more phase transitions at lower frequencies with more variability than younger adults. Based on the distinct differences in relative phase transitions to stable in-phase patterns, the authors postulated that age-related changes in the bimanual movement dynamics are function of noise in the system. As people age, neural noise increases in the areas associated with cognition and movement, and consequently the noise interferes with signal transmissions (Li et al., 2004[50]). Behavioral outcomes seen when older adults perform bimanual movements provide convincing evidence of increased noise and dysfunctional neural signal transmissions. Indeed, our two moderator variable analyses found support for this age-related conclusion in the motor impairments: (a) a large negative effect when performing asymmetrical bimanual movements and (b) increased movement variability. 

Our updated meta-analysis findings additionally confirmed that elderly adults had greater impairments in bimanual motor performances than those for younger adults. However, we need to carefully interpret these meta-analytic findings. First, all qualified studies did not separately report bimanual motor performance data between males and females. Potentially, older women may experience more structural and functional changes in the central and peripheral nervous system because of drastic changes in the sexual hormones after menopause facilitating motor deficits (Kurina et al., 2004[46]). Thus, bimanual motor impairments may be seen as different patterns of performance between older women and men. Moreover, given that the current study focused on altered interlimb motor function in the upper extremities, whether these bimanual motor impairments are observed in the lower extremities of elderly people is still unclear. For older adults, coordinating both feet is important for preventing falls during static and dynamic postural control situations (e.g., walking) (Lohman et al., 2019[52]). To clarify additional risk factors compromising independent daily activities for older adults, future meta-analysis studies should examine potential differences in bilateral motor control functions of the lower limbs between elderly and younger adults.

In conclusion, the current systematic review and meta-analysis further identified altered bimanual motor functions for older adults in comparison to those for the younger adults. Specifically, bimanual motor impairments for the elderly people increased during asymmetrical bimanual motor tasks as compared with symmetrical and complex bimanual motor tasks. Overall, older adults showed less accurate, more variable, and longer motor execution time during bimanual movement tasks. These findings suggest that rehabilitation programs for improving motor actions in older adults are necessary to focus on functional recovery of interlimb motor control including advanced motor performances as well as coordination. Potentially, bimanual movement training combined with neuromodulation techniques (e.g., non-invasive brain stimulation or neuromuscular electrical stimulation protocols) would be an option for facilitating coordinative actions in the aging motor system (Cauraugh and Summers, 2005[15]; Fried, 2022[27]; Langeard et al., 2017[47]; Sainburg et al., 2013[61]).

## Declaration

### Conflict of interest

The authors declare that they have no competing interests.

### Funding

This work was supported by a grant from the Ministry of Education of the Republic of Korea and the National Research Foundation of Korea (NRF- 2020S1A5A8041203 to NK).

## Figures and Tables

**Table 1:**
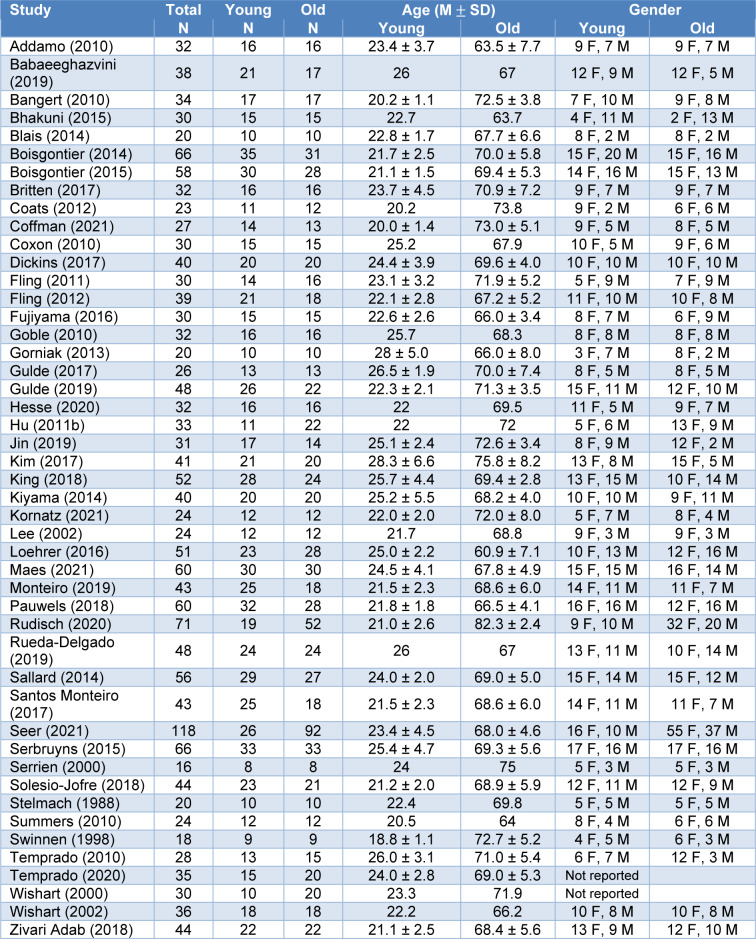
Participant characteristics

**Table 2:**
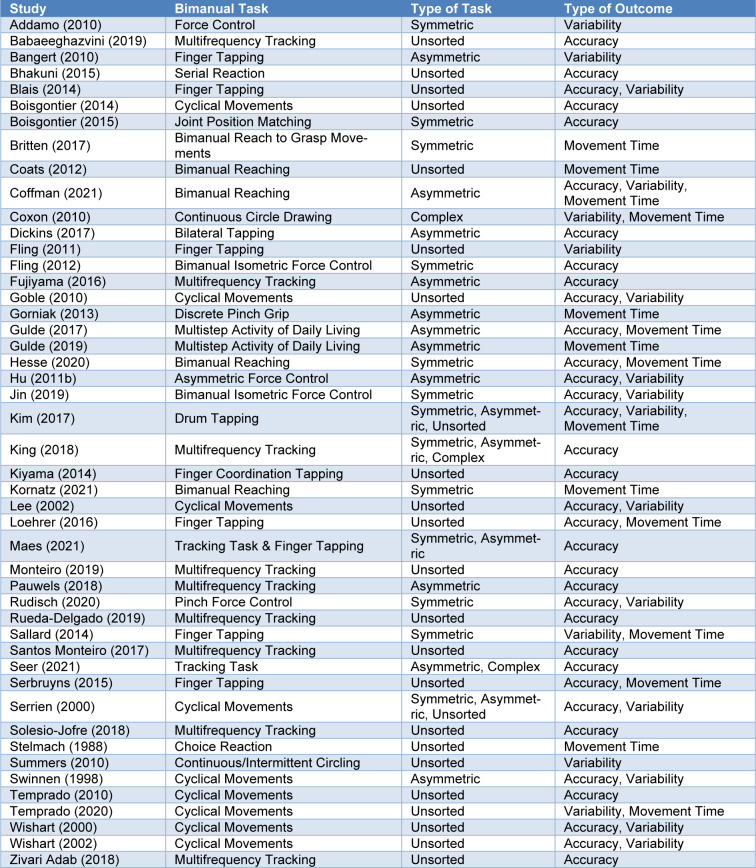
Task characteristics
